# Statistical Mechanics of Political Polarization

**DOI:** 10.3390/e24091262

**Published:** 2022-09-08

**Authors:** Miron Kaufman, Sanda Kaufman, Hung T. Diep

**Affiliations:** 1 Department of Physics, Cleveland State University, Cleveland, OH 44115, USA; 2 Levin College of Urban Affairs, Cleveland State University, Cleveland, OH 44115, USA; 3 Laboratoire de Physique Théorique et Modélisation, UMR 8089 CNRS, CY Cergy Paris University, 95031 Cergy-Pontoise, France

**Keywords:** political polarization, agent-based modeling, anticipatory scenarios, sociophysics, agent-based models, opinion dynamics, statistical physics approaches for social dynamics

## Abstract

Rapidly increasing political polarization threatens democracies around the world. Scholars from several disciplines are assessing and modeling polarization antecedents, processes, and consequences. Social systems are complex and networked. Their constant shifting hinders attempts to trace causes of observed trends, predict their consequences, or mitigate them. We propose an equivalent-neighbor model of polarization dynamics. Using statistical physics techniques, we generate anticipatory scenarios and examine whether leadership and/or external events alleviate or exacerbate polarization. We consider three highly polarized USA groups: Democrats, Republicans, and Independents. We assume that in each group, each individual has a political stance s ranging between left and right. We quantify the noise in this system as a “social temperature” T. Using energy E, we describe individuals’ interactions in time within their own group and with individuals of the other groups. It depends on the stance s as well as on three intra-group and six inter-group coupling parameters. We compute the probability distributions of stances at any time using the Boltzmann probability weight exp(−E/T). We generate average group-stance scenarios in time and explore whether concerted interventions or unexpected shocks can alter them. The results inform on the perils of continuing the current polarization trends, as well as on possibilities of changing course.

## 1. Introduction

Polarization consists of “a process of alignment along multiple lines of potential disagreement and measured as growing constraint in individuals’ preferences” [1]. According to [2], polarization results from individuals forming “broad and encompassing clusters organized around cohesive packages of beliefs”. Political polarization—“the increasing ideological distance between the left and the right” [3]—is accompanied by affective polarization, which is a dislike and distrust of individuals belonging to the opposing political party [4]. This further reduces inter-group exchanges and enhances homophily, defined as “a greater interaction between like-minded individuals”, which can itself be a source of polarization [5,6].

**Figure 1:**
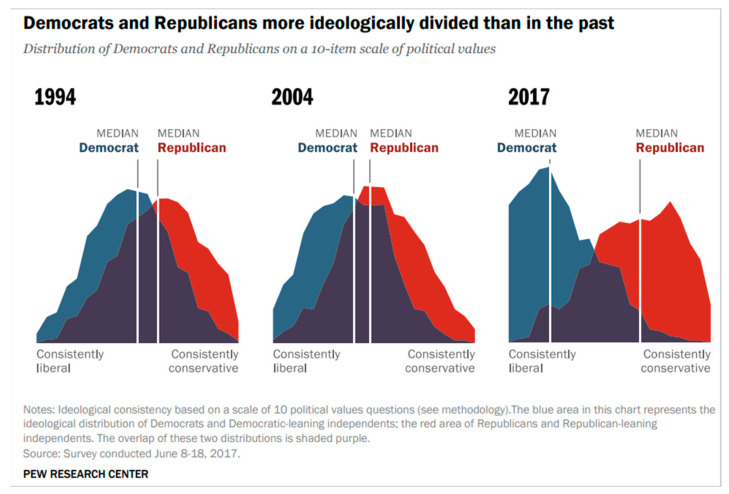
Growing Democrat–Republican division, 1994–2017 [7].

The United States is increasingly and sharply polarizing politically ([7,8,9,10,11,12]; Figure 1) but is not alone in this [13] and, according to polls, people see it [10]. This trend in the American public began in the 1970s [14]. It is mirrored by media polarization, with each side only trusting the media favoring their own side [15]. The political differences tend to revolve around a set of issues including government aid to the needy, race, immigration, national security, and the environment [7].

Political polarization has serious societal [1,16,17,18] and economic [3] consequences. It harms democracies [16,17,18]. Unchecked, it gradually defeats people’s ability to make joint decisions and slowly leads to societal breakdown as individuals no longer see any commonality, joint objectives, or reasons to interact with any but those who share their political views [18]. While, in general, interactions between people might be expected to reduce their differences [19], lack of inter-group interactions (homophily) deepens differences to the detriment of society. Polarization fosters a Manichaean (good–bad) outlook on issues, with no gray areas and no attention to the complexity of reality [18], and increases the likelihood of social conflict [19]. One casualty of polarization is truth: polarized individuals expect scientific information to align with their political perspective, regardless of its factual basis [18,20,21]. Another downside of polarization is that needed change can no longer occur through debate, give-and-take, and joint decisions, and instead tends to occur by fiat when one side accumulates sufficient power to impose it, only to be undone when the other side takes a turn in power [18]. Given the socially destructive consequences of polarization, we need to anticipate its possible direction and magnitude in time, and explore interventions. In other words, we need to “confront surprise” [21]. While this is impossible in practice, we can at least prepare information that might be useful in facing polarization [21,22,23,24].

Polities, however, are complex systems [25] with many interdependent moving parts, hinging on initial conditions, and with hard-to-identify causes and their links to effects, especially over time. According to [26,27], empirical studies do not suffice to help us understand political polarization dynamics, which require theoretical modeling. Agent-based modeling has great potential in this regard [26,27]. Although scholars from several disciplines have been studying political polarization for several decades, sociophysics, which involves applying physics tools to the study of social phenomena, has been the cutting edge of models which can handle complexity in various domains, including politics. They provide insights complementing those gleaned from other disciplines [28,29].

One sociophysics approach for modeling complex systems uses mean-field models [23,30,31,32,33]. Such models have been called “generative” as opposed to inductive or deductive and are arguably well suited to assist decision making [34]. They have already been specifically used in studies of polarization e.g., [9,12,19,35,36,37,38]. Despite the seeming simplicity of concept (compared to the traditional, complicated, and multi-variate regression models prevalent in social sciences), such models allow for the testing of hypotheses regarding issues such as the role of the media in social dynamics e.g., [33] or the use of bot agents to alter public opinion [39].

Mean-field models can be used to explore polarization trends, find avenues for intervention, and avert some of its negative consequences. Since prediction in the context of a complex system is at best limited [25], generating qualitative anticipatory scenarios that can be queried e.g., [40,41,42,43,44,45] is an alternative. This entails producing a range of possible futures with respect to a variable of interest, mapping their consequences, and exploring intervention possibilities. The general mean-field approach has gained currency in the first decades of the century as scholars applied it to a variety of contexts. For example, using mean-field models, we anticipated election outcomes in the US and in Bosnia–Hercegovina [28,42], and various labor-management contract negotiations results [45]. As [46] argued, anticipatory scenarios are useful in supporting the development of robust strategies of action in the face of high levels of uncertainty in characterizing complex systems.

We take this approach in anticipating the course of political polarization in the United States. We propose a three-group model to represent Democrats, Republicans, and Independents. Informed by public-poll results, we qualitatively estimate the model’s parameters and reproduce the polarization pattern in time, as reflected in the polls. We explore effects of leadership and of a focusing event on the polarization trends. We find that leadership has a transitory effect in reducing polarization, while a focusing effect has the potential for a more lasting impact in bringing people together, but it cannot be controlled.

## 2. Model

We extended a sociophysics two-group mean-field model of conflict dynamics [40] to three political groups in the US: Democrats (group 1), Republicans (group 2), and Independents (group 3). Groups 1 and 2 drive the polarization, often measured as the average gap between them. Group 3 matters, however, because, considering 2004 Independents have represented between 27% and 50% of the population [47], they are recruitment pools for the other two groups and gain importance especially at election times.

We considered each group to be a network of individuals, with each vertex connected to all others with bonds of equal intensity [40,42]. The couplings do not depend on the distance between individuals. This is an equivalent-neighbor or mean-field model.

In each group, each individual has a stance $s_{i}$ regarding a specific issue under debate, i.e., economics, social issues, defense, etc., or (as described here) a package of such issues (in the [1,6] sense). The stance $s_{i}$ varies between −1 and +1, where −1 corresponds to the Democrats/progressive/left position, while +1 corresponds to the Republicans/conservative/right position. Individuals align with the group whose average stance is closest to their own [1]. Individuals inside a group are homophilic [5], i.e., they tend to prefer to communicate with each other rather than with individuals from a different group.

We note that in our previous work on two- and three-group conflicts [40,41,42,43], the stance $s_{i}$ was discrete. Here, the stances $s_{i}$ are continuous, which we believe is more realistic and eliminates the arbitrariness of selecting a discrete number of states.

We computed the average stance s of each group using the Boltzmann probability distribution exp(−E/T). The negative energy associated with an individual in group 1 is $-E_{i}={\;\mathrm{Js}}_{i}s1+{\;\mathrm{Hs}}_{i}$, where $s_{i}$ is the stance of that individual and s1 is the mean of all stances in group 1. The coupling J quantifies the cohesiveness of the group. The magnetic field H represents the (possible) action of the group’s leadership on the individuals: for H > 0, the mean stance is pushed to positive values, while for H < 0, the mean stance is pushed to negative values. For group 1:

(1) $$s1_{t+1}=\frac{\;\int_{-1}^{1}{\mathrm{se}}^{-\frac{\mathrm{Js}1_{t}s+\mathrm{Hs}}{T}}\mathrm{ds}\;}{\int_{-1}^{1}e^{-\frac{\mathrm{Js}1_{t}s+\mathrm{Hs}}{T}}\mathrm{ds}}$$

We denote j = J/T, h = H/T. The result of the integration (1) can then be expressed with the Langevin function (which is used to model paramagnetic materials):

(2) L(x)=cotanh(x)−1/x

Then, Equation (1) becomes

(3) $$s1_{t+1}=L\left(\mathrm{js}1_{t}+h\right)$$

The average stance s at time t+1 is assumed to be determined by preferences of the group at an earlier time t. This lag represents the time it takes to change individuals’ stance, for instance, by peers’ persuasion. The time t is expressed in units of the lag time.

Within each group, members try to persuade each other to their own stance, thereby reducing the intra-group stance differences. They also take account of the other groups’ average attitudes. In time, average group attitudes evolve depending on the values of intra-group cohesion parameters J and the inter-group influence parameters K. For example, that is how Democrats and Republicans attract Independents into their respective groups since their internal cohesion J3 is nil, i.e., they are not a formal group.

In the presence of two other groups, we assumed that the energy function L of group 1 contains two added interaction terms, i.e., $K12s2_{t}+K13s3_{t}$, representing the influence of the mean stances of groups 2 and 3, respectively, on an individual i in group 1. Thus, for each of the three groups,

(4) $$s1_{t+1}=L\left(h1+j1s1_{t}+k12s2_{t}+k13s3_{t}\right)\;s2_{t+1}=L\left(h2+j2s2_{t}+k21s1_{t}+k23s3_{t}\right)\;s3_{t+1}=L\left(h3+j3s3_{t}+k31s1_{t}+k32s3_{t}\right)$$

Here, h1 represents H1/T, k12 represents K12/T, etc. The inter-group interactions K12 and K21 are not necessarily equal. At times, members of one group may feel cooperative toward the other, who might not reciprocate.

We define a polarization measure as the distance between the mean stances of groups 1 and 2 at any time:

P = (s2 − s1)/2(5)

It is defined so that −1 ≤ P ≤ 1. The unpolarized case P = 0 corresponds to equal stances, i.e., s1 = s2. Polarization is extreme when P = 1, corresponding to the Republicans’ stance s2 = 1 (conservative/right) and Democrats’ stance s1 = −1 (progressive/left), and when P = −1, corresponding to the Republicans’ stance s2 = −1 and Democrats’ stance s1 = 1.

The model has twelve parameters: three for the groups’ respective internal cohesiveness J, six representing how they relate to each other, K, and three H. They can be estimated qualitatively, as we have done here, informed by publicly available poll data (here, we used [7,41,48]).

## 3. Results: Scenarios of Polarization

To produce anticipatory scenarios of polarization, we made the following assumptions:

- The Democrats (group 1) are more cohesive than Republicans (group 2), i.e., J1 > J2;
- Independents (group 3) have no cohesion (J3 = 0) because they have no structure or means of identifying with each other, do not communicate, and do not recruit; therefore, they exert no influence on the other two groups and, as such, K13 = K23 = 0;
- Independents tend to be contrarian to the party in power (here, group 1), thus K31 < 0, and are not influenced by the opposition party, thus K32 = 0 (see [49,50,51] for other examples of contrarian use in a model).

For the scenarios described next, we kept intra-group couplings fixed. We distinguished three qualitatively different situations depending on K12 and K21, which are the interactions between groups 1 and 2. Scenario A is competitive in that K12 < 0 and K21 < 0 (Democrats and Republicans are contrarian). Scenario B is mixed in that K12 > 0 and K21 < 0 (lack center, while Republicans remain contrarian). Scenario C is collaborative in that K12 > 0 and K21 > 0 (both Democrats and Republicans are open to working together), perhaps driven by a focusing crisis. The intra-group coupling parameter values we chose to be consistent with our assumptions are J1 = 5, J2 = 3, and J3 = 0.

**Figure 2:**
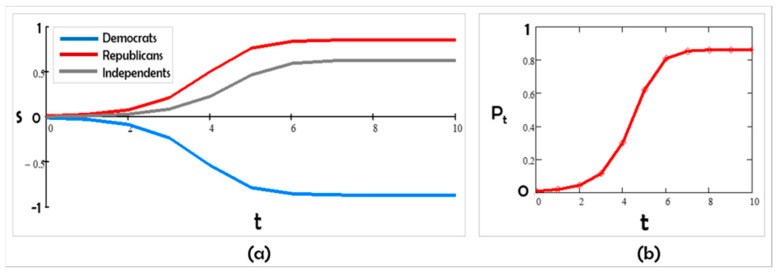
Scenario A: (**a**) s trajectories with competitive K12 < 0, K21 < 0, and K31 < 0, and (**b**) the corresponding polarization in time.

In Scenario A, Democrats and Republicans are mutually contrarian. Even when starting with all three groups’ positions close to one another, over time, the system polarizes and remains polarized (Figure 2). The Independents, whom we assumed to be contrarian to the party in power (here, Democrats), track the Republicans’s values. This scenario matches the trends found by the Pew polls of 1994-2017 ([7]; Figure 1) and anticipates that they might continue unabated. The group parameter values we chose to be consistent with our assumptions are

J1 = 5, J2 = 3, J3 = 0, K12 = −4, K21 = −5, K31 = −3, and K13 = K23 = K32 = 0

J1 = 5; J2 = 3; J3 = 0; K12 = −4; K21 = −5; K31 = −3; and K13 = K23 = K32 = 0

**Figure 3:**
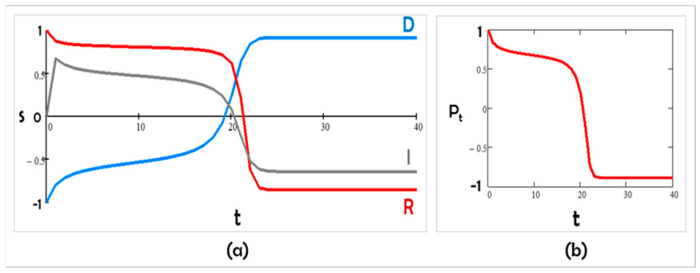
Competitive scenario: (**a**) K12 < 0, K31 < 0, and added leadership effect (magnetic field H1 > 0), and (**b**) the corresponding polarization in time.

We wondered how Scenario A would change if the internal cohesions and inter-group parameters remained unchanged but a political leader intervened to push Democrats to become conciliatory (Figure 3). Here, we simulated the leadership of group 1 (Democrats) acting to move s1 closer to group 2 (H1 > 0). We illustratively chose H1 = 4. For a strong enough value of the magnetic field H, polarization, which began as positive, changed over time, passing through zero and ending in the negative range. We could read this as effective intervention up to a point (since polarization goes to 0) but its effect in time is limited and eventually the parties slide into their oppositional stances. The model allows for an exploration into how the “harmony” period might be extended.

J1 = 5; J2 = 3; J3 = 0; K12 = −4; K21 = −5; K31 = −3; K13 = K23 = K32 = 0; and H1 = 4

**Figure 4:**
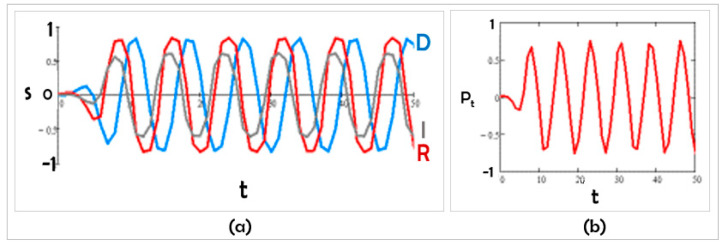
Scenario B: (**a**) K12 > 0, K21 < 0, and K31 < 0, and (**b**) the corresponding polarization in time.

In Scenario B, we considered a mixed situation: Democrats are collaborative while Republicans remain contrarian; thus, K12 > 0, K21 < 0, and K31 < 0. We chose K12 = 4, K21 = −5, K31 = −3, and K13 = K23 = K32 = 0. This could occur, for example, if Democrats lost power and needed Republican help to pass bills. Then, the stances s1, s2, and s3, and the polarization P displayed oscillations (Figure 4).

J1 = 5; J2 = 3; J3 = 0; K12 = 4; K21 = −5; K31 = −3; and K13 = K23 = K32 = 0

**Figure 5:**
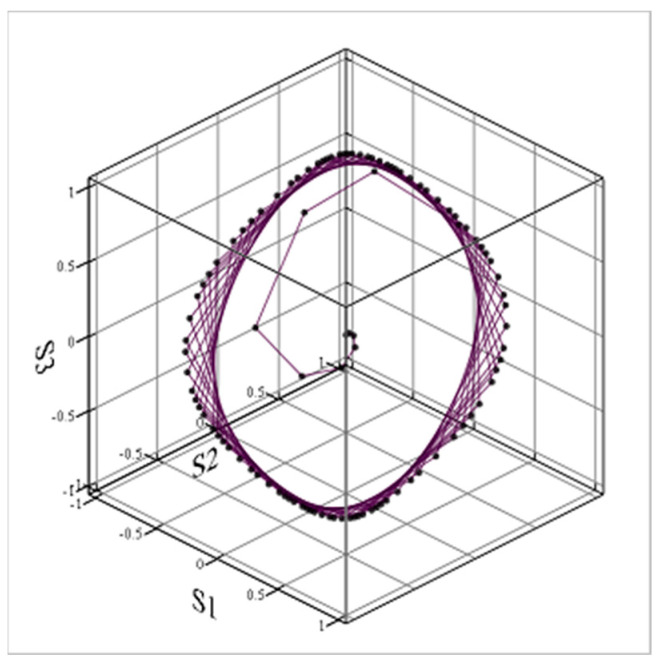
Phase-space limit cycle of stances in the mixed scenario.

In the three-dimensional space of stances (s1, s2, and s3; Figure 5), the system trajectory is a limit cycle.

J1 = 5; J2 = 3; J3 = 0; K12 = 4; K21 = −5; K31 = −3; and K13 = K23 = K32 = 0

**Figure 6:**
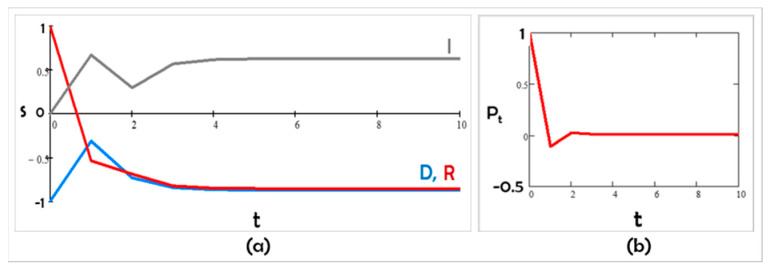
Scenario C is cooperative scenario: (**a**) K12 > 0, K21 > 0, and K31 < 0, and (**b**) the corresponding polarization in time.

Scenario C is a collaborative case where K12 and K21 are both positive. To illustrate this, we chose K12 = 4, K21 = 5, K31 = −3, and K13 = K23 = K32 = 0. As a result, s1 and s2 became closer to each other over time, i.e., the polarization vanished (Figure 6). The Independents, in this case, are separated from both Democrats and Republicans, i.e., s3 > 0 and s1 ≃ s2 < 0, due their contrarian stance to power reflected in the coupling K31, which is negative. Such a scenario, unlikely as it may seem at the height of political polarization, might emerge if the country were to face a “focusing” crisis. In the past, wars have had this effect. Not all crises are unifying, however. The COVID-19 pandemic, for example, deepened differences over science, policy, and politics.

J1 = 5; J2 = 3; J3 = 0; K12 = 4; K21 = 5; K31 = −3; and K13 = K23 = K32 = 0

Since our parameters are only qualitatively informed by poll data (in the sense of consistency with the poll results of Figure 1, which show a relatively rapidly increasing gap between Democrats and Republicans), we checked how sensitive our anticipated stance trajectories are to errors in the estimates. We found that the predictions react only slightly to small changes (perturbations) in the coupling values. Thus, this model is robust with respect to small perturbations. This means that estimation errors do not cause a qualitative change in the scenarios. To illustrate this, we calculated the change in s1 at time t+1 due to a small change in the intra-group coupling j1. We used Equation (4) and found that

(6) $$\delta s1_{t+1}=\frac{\mathrm{dL}}{\mathrm{dx}}s1_{t}\delta j1$$

where x represents $h1+j1s1_{t}+k12s2_{t}+k13s3_{t}$.

The derivative of the Langevin function, as defined in Equation (2), is

(7) $$\frac{\mathrm{dL}}{\mathrm{dx}}=1-\cot\mathrm{anh}{\left(x\right)}^{2}+\frac{1}{x^{2}}$$

We can show that $0<\frac{\mathrm{dL}}{\mathrm{dx}}\;\leq \frac{1}{3}$. Hence, from Equation (6), we found that the fractional error in s1 is bounded; thus,

(8) $$|\frac{\delta s1_{t+1}}{s1_{t}}|\;\leq \frac{1}{3}|\delta j1|$$

**Figure 7:**
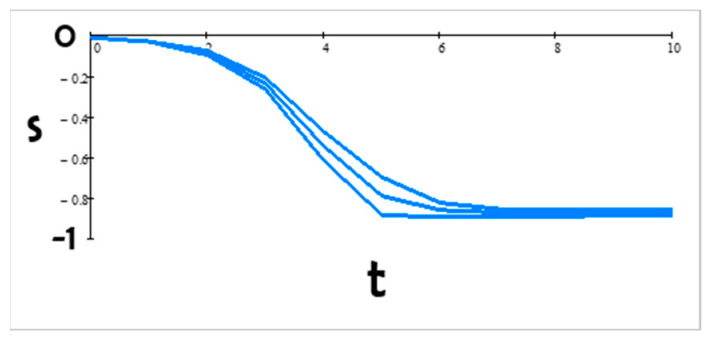
Robustness of model-stance (here, Democrat) trajectory to small perturbations of the parameter values in time.

In Figure 7, we show the time dependence of s1–δs1, s1, and s1 + δs1 for ∣δj1∣ = 1. The three curves are close to each other, demonstrating that the model is robust to small perturbations.

## 4. Discussion

Our results are both methodological and substantive.

Methodologically, we anticipated the progression of polarization using a sociophysics mean-field model. Anticipating a range of possible futures is useful and necessary for policy makers; they need to make robust decisions likely to work for the range rather than rely on one predicted future that may not materialize. We have illustrated how to generate qualitative anticipatory scenarios informed by publicly available poll data. We also showed that these scenarios are not sensitive to small parameter changes (Figure 7). We have shown how we can query the original scenario (Figure 2) for effects of leadership (Figure 3) and focusing events (Figure 6). We plan to further enhance this model by adding to the number of interacting groups and by relaxing the current assumption that each individual interacts with every other individual in the system; for example, interactions could go through central figures or leaders. We also plan to explore changes in the intergroup parameters in time.

Substantively, the first scenario we obtained (Figure 2) based on our assumptions is similar to the Pew poll results of 1994–2017 ([7]; Figure 1). According to [52], the ability to recreate patterns that match data (such as polls) is a measure of understanding the phenomenon we are studying using agent-based modeling; “If you didn’t grow it, you didn’t explain it” [34].

We plan to improve the model in several ways. We will test the effect of K values for the Independents that create the “leaning Democrat” and “leaning Republican” subsets that exist in reality. We will also combine leadership and focusing events since leadership can make a considerable difference at times of crisis. We plan to explore the ways in which the intra- and inter-group parameters could change in time in reaction to internal group dynamics or external events. Since context plays an important role in real situations, we intend to explore its effects on polarization. Finally, we plan to examine scale effects e.g., [52] and explore the conjecture in [53] that the local convergence of stances can generate global polarization. We will investigate, using Monte Carlo simulations, the role of the range of interactions among the individuals of any given group.

## 5. Conclusions

We have investigated political polarization, which is rising around the world and impeding polities from making necessary joint decisions. Numerous scholars are looking into its antecedents and its consequences. We are contributing a tool for gaining insights into future directions and into possible effects of intervention (e.g., through leadership), or under the effect of focusing events, e.g., natural disasters or wars.

The tool we propose is a dynamic sociophysics model which has proven effective in addressing the complexity of social systems, as well as demonstrated to be versatile in its adaptability to several very different contexts, from political contests to regional economics, international disputes, and labor relations. While it is qualitative, it draws on publicly available data, such as opinion polls, and is relatively parsimonious in a number of parameters, especially considering the numerous variables likely affecting the phenomena studied. We used it, as illustrated in the case of polarization, to generate anticipatory scenarios that allow us to ask “what if” questions, such as “what if” a conciliatory leader emerged in one group, would polarization diminish? This practice is expanding in domains such as urban planning and business because it offers insights about a system that are difficult to gain by other approaches.

## Data Availability

Not applicable.
